# Supplementary material for: A whole-animal phenotypic drug screen identifies suppressors of atherogenic lipoproteins
Source: eLife. 2026 Aug 27;14:RP105314. doi: 10.7554/eLife.105314 (PMC13521566; doi:10.7554/eLife.105314)
Supplement: Supplementary file 2 [file elife-105314-code1.zip › JHDL_primary_result/FENBENDAZOLE/FENBENDAZOLE_JHDL_7_033_003_2019_06_14_11_10.pdf]

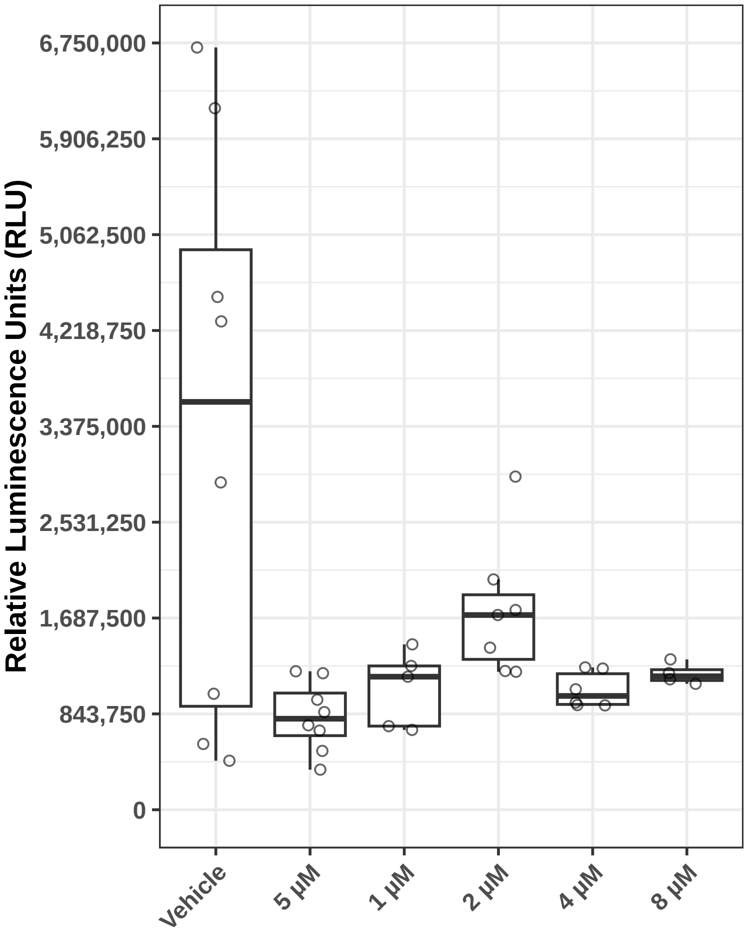

Relative Luminescence Units (RLU)
6,750,000
5,906,250
5,062,500
4,218,750
3,375,000
2,531,250
1,687,500
843,750
0
Vehicle
5 μM
1 μM
2 μM
4 μM
8 μM
